# Plankton classification with high-throughput submersible holographic microscopy and transfer learning

**DOI:** 10.1186/s12862-021-01839-0

**Published:** 2021-06-16

**Authors:** Liam MacNeil, Sergey Missan, Junliang Luo, Thomas Trappenberg, Julie LaRoche

**Affiliations:** 1 Biology Department, Dalhousie University, 1355 Oxford Street, Halifax, NS B3H 4J1 Canada; 2 4Deep inwater imaging, 71 Appaloosa Run, Hammonds Plains, NS B4B 0G2 Canada; 3 Department of Computer Science, Dalhousie University, 6050 University Avenue, Halifax, NS B3H 4R2 Canada

**Keywords:** Holographic microscopy, High-throughput imaging, Deep learning, Convolutional neural networks, Plankton, Classification workflow, Deployable microscope

## Abstract

**Background:**

Plankton are foundational to marine food webs and an important feature for characterizing ocean health. Recent developments in quantitative imaging devices provide in-flow high-throughput sampling from bulk volumes—opening new ecological challenges exploring microbial eukaryotic variation and diversity, alongside technical hurdles to automate classification from large datasets. However, a limited number of deployable imaging instruments have been coupled with the most prominent classification algorithms—effectively limiting the extraction of curated observations from field deployments. Holography offers relatively simple coherent microscopy designs with non-intrusive 3-D image information, and rapid frame rates that support data-driven plankton imaging tasks. Classification benchmarks across different domains have been set with transfer learning approaches, focused on repurposing pre-trained, state-of-the-art deep learning models as classifiers to learn new image features without protracted model training times. Combining the data production of holography, digital image processing, and computer vision could improve in-situ monitoring of plankton communities and contribute to sampling the diversity of microbial eukaryotes.

**Results:**

Here we use a light and portable digital in-line holographic microscope (The HoloSea) with maximum optical resolution of 1.5 μm, intensity-based object detection through a volume, and four different pre-trained convolutional neural networks to classify > 3800 micro-mesoplankton (> 20 μm) images across 19 classes. The maximum classifier performance was quickly achieved for each convolutional neural network during training and reached F1-scores > 89%. Taking classification further, we show that off-the-shelf classifiers perform strongly across every decision threshold for ranking a majority of the plankton classes.

**Conclusion:**

These results show compelling baselines for classifying holographic plankton images, both rare and plentiful, including several dinoflagellate and diatom groups. These results also support a broader potential for deployable holographic microscopes to sample diverse microbial eukaryotic communities, and its use for high-throughput plankton monitoring.

**Supplementary Information:**

The online version contains supplementary material available at 10.1186/s12862-021-01839-0.

## Background

Plankton are an integral component of the global ocean. Plankton abundance and composition can be coupled to environmental conditions and yield important insights into aquatic food webs (e.g., [1, 2]). Often hugely diverse and occupying numerous trophic modes in surface ocean ecosystems, classifying plankton composed in a water mass is challenging, error prone, and a bottleneck of time and costs. Recent developments in imaging instruments allow biological contents to be visualized directly from bulk volumes at high image resolution, without disintegrating cell structures [3]. Imaging instruments have used a variety of optical methods including flow cytometry [4], shadowgraphs [5], holography [6], among others. Several such devices have imaged plankton size classes that collectively encompass autotrophs and heterotrophs, spanning four orders of magnitude in size from 2 μm to 10 cm [3, 7, 8]. The high sampling frequency from digital imaging also opens new ecological challenges exploring microbial eukaryotic diversity [9], alongside technical challenges to automate classification from spatial and temporally dense datasets (e.g., [10, 11]).

Digital holography is based on the diffracted light field created by interference from objects in a sample which is illuminated by a coherent light (e.g., a laser): That interference pattern is recorded by a digital sensor and composes a hologram [12]. Since their inception [13], holographic microscopes have been applied widely at micrometre scales to observe, for example, particle distributions [14], coral mucus production [15], and to differentiate cancerous pancreatic cells from healthy ones [16]. Holographic microscopes have advanced considerably with improving computational techniques for digital reconstruction and focus enhancement [17]. Digital in-line holographic microscopy (DIHM) with a point-source laser is a simple, lens-free implementation of Gabor-style holography that can capture a 3-D sample using a common path optical configuration, whereby both reference and interfered light waves copropagate and are recorded by a digital camera [18]. DIHM has several advantages for biological studies including a simple design with a larger depth of field than conventional light microscopy, allowing rapid imaging of larger volumes and 3-D numerical refocusing with no required staining of cells [19, 20]. Due to its simplicity, DIHM can easily be incorporated into various cell imaging configurations including amplitude and phase images [21] and to date, numerous studies have used holography to image marine plankton [8, 22–25]. There is increasing interest to use its advantages towards automating classification of plankton and particulates from water samples (e.g., [26–28]). A review of holographic microscopes for aquatic imaging can be found in Nayak et al. [29].

Plankton exhibit substantial morphological variation within and between major groups, are often imaged at different orientations, appear partially occluded, or damaged. Extracting features from plankton images originally relied on handcrafted feature descriptors, which are label-free and train classifiers like support vector machines or random forest efficiently [30]. But detecting features based on predefined traits rapidly reaches its limits. Instead, deep learning algorithms have gained popularity for their state-of the-art performance and, at least in part, because they require no domain specific knowledge or impose descriptors for pattern recognition, rather features are learned during training [31]. Deep learning involves representing features at increasing levels of abstraction and for image tasks, the most successful models have been convolutional neural networks (CNNs): a layered neural network architecture, with layers equating to depth, and where convolutions substitute as feature extractors [32]. These CNNs learn features through sequential layers connected to the local receptive field of the previous layer and the weights learned by each kernel [32]. For plankton, CNNs have improved the classification stage of automation efforts [33, 34]. But the natural imbalance in plankton datasets and frequent drifts in class distributions [35] render accuracy benchmarks for performance biased towards majority classes and poor evaluation metrics (e.g., [36, 37]).

Achieving state-of-the-art classification at scale often requires large training datasets for CNNs, but generic features can be extracted from pre-trained models and repurposed—termed transfer learning—such that CNNs have a baseline that can recognize features unspecific to any image, similar to Gabor filters or color blobs [38]. Transfer learning has achieved classification benchmarks equivalent to traditional feature descriptors (e.g., [39]). Large plankton image datasets do exist—some containing several million labelled images across hundreds of classes (e.g., [40])—but there is a current lack of easily deployable plankton imaging devices capable of rapidly sampling several litres. Holographic microscopy combined with computer vision, could bridge high throughput in-situ data production with increasingly automated classification and enumeration of major plankton groups.

The purpose of this study is to show whether species of micro-mesoplankton can be detected in-focus from volumetric samples, classified with deep learning algorithms, and to evaluate classifiers with threshold-independent metrics—which, to date, are rarely considered for imbalanced plankton classification tasks.

## Methods

### The HoloSea: submersible digital in-line holographic microscope (DIHM)

General DIHM designs for biological applications are reviewed in Garcia-Sucerquia et al. [6] and Xu et al. [20]. A similar submersible DIHM, the 4-Deep HoloSea S5^1^ (92 × 351 mm, 2.6 kg), first introduced by Walcutt et al. [41], was used here to image plankton cells. Its principal advantage is a simple lensless, in-flow configuration with 0.1 mL per frame and high frame rates (> 20 s^−1^) that support a maximum flow rate > 130 mL min^−1^. Housed in an aluminum alloy casing, the HoloSea uses a solid-state laser (405 nm) coupled to a single mode fiber optic cable acting as a point source to emit spherical light waves through a sapphire window. As light waves travel through the sampled volume, both the waves scattered by objects and reference waves copropagate until they interfere at the plane of the monochrome camera sensor (CMOS) to form an interference pattern (i.e., a 2048 × 2048 hologram). The camera is aligned 54 mm away from the point source and recorded holograms are stored as PNG images for further numerical reconstruction and analyses.

### Numerical hologram reconstructions

**Fig. 1:**
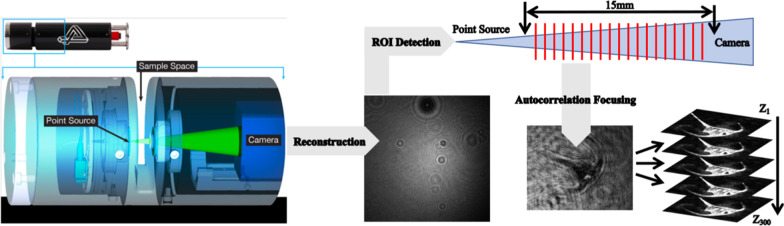
The workflow for imaging, detecting, and selecting in-focus objects. Volumes are recorded in the microscopes sample space and the interference pattern is reconstructed to create a hologram. Plankton objects are first detected as ROIs across 300 reconstructed planes (i.e., z-distances) of a hologram corresponding to the 15 mm sample space. The plane containing an in-focus object is calculated via autocorrelation and Vollath’s F4 algorithm

Hologram reconstruction from point-source holography was first proposed by Kreuzer et al. [42], and its principles are well described [19–21]. The workflow from reconstruction to object focusing are shown in Fig. 1. In order to recover the information about objects within holograms at the specific focal distance from the point source, wave front intensity was digitally reconstructed based on a Helmholtz-Kirchhoff transformation [43] in 4-Deep Octopus software.^2^ Each hologram was reconstructed at multiple z-distances from the point source using a 50 μm step size through the sample volume. To detect regions of interest (ROIs) in each reconstructed plane, we used 4-Deep Stingray software^3^ with a globally adaptive threshold algorithm based on Otsu [44]. During the detection step, ROIs could also be discriminated based on their size, for our purposes, we defined a range of two orders of magnitude (20–2000 μm) to encompass micro-mesoplankton. Detected ROIs were clustered together across multiple z-planes based on the Euclidian distances between their centroids using the Density Based Spatial Clustering with Applications of Noise (DBSCAN) algorithm [45]. Each resultant cluster contained the same ROI tracked at multiple consecutive z-planes within the volume. To identify the plane containing an in-focus object within each cluster, we used Vollath’s F4 autocorrelative algorithm [46]—the object with the highest correlation score between pixels was then stored in our database and the rest of objects within the cluster were discarded.

### Holographic image dataset

The plankton for our experiments (Table 1) included monocultures grown in artificial seawater and 500 mL surface (1 m) water samples from Bedford Basin compass buoy station (44° 41′ 37″ N, 63° 38′ 25″ W). Monoculture samples were grown under f/2 nutrient replete and the recommended temperature and light conditions (Bigelow Laboratories, Maine, USA). Samples were pumped through the sample chamber using a peristaltic pump and recorded at 10 fps. The resulting image dataset was augmented by rotating each image horizontally, vertically, and translated to enlarge the number of training images, and hence the learnable features threefold [47]. All images were scaled to 128 × 128 pixels preserving the aspect ratio of the source images. Classes were randomly split approximately 50:10:40 for training, validation, and testing, respectively. Training and validation samples were divided into five stratified k-folds, where each fold retains the proportion of classes in the original training set [48]. We included a “noise” class to filter holographic artefacts [49].

**Table 1:** Taxa identity, size ranges, and total number of images

Class	Taxonomic group	Size (µm)	Strain	Examples
*Alexandrium tamarense*	Dinoflagellate	20–80	CCMP1771	201
*Ceratium fusus*	Dinoflagellate	50–350	Environmental	56
*Ceratium lineatum*	Dinoflagellate	80–230	Environmental	44
*Ceratium longpipes*	Dinoflagellate	200–340	CCMP1770	378
*Ceratium* sp.	Dinoflagellate	140–230	Environmental	64
*Chaetoceros socialis*	Diatom	40–360	CCMP3263	102
*Chaetoceros* straight	Diatom	30–120	CCMP215	325
*Chaetoceros* sp.	Diatom	30–430	CCMP1690	114
Crustacean	Animal	180–640	Environmental	13
*Dictyocha speculum*	Silicoflagellate	30–105	CCMP1381	185
*Melosira octagona*	Diatom	80–460	CCMP483	173
Noise	Artefact	–	–	150
*Parvicorbicula socialis*	Choanoflagellate	25–85	Environmental	36
*Prorocentrum micans*	Dinoflagellate	30–120	CCMP688	1074
*Pseudo-nitchzia arctica*	Diatom	35–150	CCMP1309	33
*Rhizosoenia setigera*	Diatom	200–530	CCMP1330	306
Rods	Morphological	60–280	–	396
*Skeletonema costatum*	Diatom	60–130	CCMP2092	157
Tintinnid	Ciliate	90–310	Environmental	20

Cell sizes are taken from apical cell length measurements, using 25 examples for each class

### Convolutional neural networks (CNNs)

The plankton detected in our holograms were classified with four different CNNs: VGG16 [50], InceptionV3 [51], ResNet50V2 [52], and Xception [53]. In terms of model depth, VGG16 is the shallowest, InceptionV3 and ResNet50 are near equal, while Xception is the deepest. Each uses convolutions as feature extractors but with different model architecture (See Additional file 1: Table S1). Due to the modest size of our plankton dataset, we used a transfer learning approach where each model was pre-trained on ~ 1.4 M images binned into over 1000 classes from the ImageNet dataset [54]. Pre-trained models have already learned generalizable features from the ImageNet dataset—which includes animals, sports objects, computers, and other classes very different from plankton—that provides a powerful baseline for feature recognition [38]. Classification was implemented in the Python deep learning toolbox Keras [53], which is accessible as a core component of the Tensorflow package [55].

Each model was applied in two different ways, first as a feature extractor by only retraining the deepest model layers to preserve the pre-tuned weights [38], and secondly by maintaining the first 10–20 layers and retraining the remaining layers. The second method was exploratory and involved freezing the first 10 layers in VGG16, and the first 20 layers for the other deeper models, which have presumably already learned generic features. We used dropout for each method at a probability of 0.3 to prevent overfitting [56] and added a Softmax classifier to transform the fully connected vector into a probability distribution specific to 19 classes [57]. Our images were preprocessed according to each CNNs requirements [58], and the greyscale color channel was repeated for each colored channel (i.e., RGB) that the models observed from ImageNet.

Prediction bias from our class imbalances, where the most abundant class was nearly three times greater than the least abundant, was offset by maintaining class proportions during training using stratified k-folds [59]. Combining the predictions on the validation and test sets from each fold, for each model, created an ensemble of networks to evaluate prediction variance [60]. Training was repeated for 20 epochs for each fold, where an epoch represents an entire pass of the training set. Training specifications included a batch size of 32, and momentum values of 0.9 in batch normalization layers of ResNet50, InceptionV3, and Xception [61]. The learning algorithm minimized the log loss (cross-entropy) function through backpropagation using the Adam optimizer [62]—the learning rate was set at 0.01 and reduced by a factor of 10 if the loss function failed to improve by 1e^−3^ after five epochs. Holographic reconstructions, object detection and classification were implemented in the NVIDIA CUDA GPU toolkit [63] using a NVIDIA GeForce GTX960 GPU with 16 GB of RAM.

### Validation measures

Classification performance was evaluated using three broad families of metrics: Thresholding, probabilistic, and ranked. To extend each metric to our multi-label problem, we binarized classes (one vs. all) to mimic multiple binary classification tasks. Thresholding measures are estimated from the quantity of true positives ($tp$), true negatives ($tn$), false positives ($fp$), and false negatives ($fn$) observed during training and testing. These measures assume matching class distributions between training and test sets, which we satisfied in each stratified fold. Accuracy is simply defined by the total proportion of correct predictions, whereas precision is defined by the proportion of correctly predicted positives ($tp$) to all predicted positives ($tp+fp$), also known as the predictive positive value (1).

1 $$Precision=\frac{tp}{tp+fp}$$

The recall defines the proportion of correctly predicted positives ($tp$) to all positive examples ($tp+fn$), it is equivalent to the true positive rate (2).

2 $$Recall \left(Sensitivity\right)= \frac{tp}{tp+fn}$$

The balanced score between precision and recall can be represented by the F1-score, calculated using a harmonic mean (3) [64].

3 $$F1= 2*\frac{\left(Precision*Recall\right)}{\left(Precision+Recall\right)}$$

Ecologically meaningful plankton classifiers predict few false positives and a high proportion of true positives across all classes [65]. This priority favors precision, because high precision scores imply few false positives, and the F1-score as the relative balance between precision and recall, as such, high F1-score contains fewer false positives and false negatives across all labels [65]. Although both metrics are more sensitive than accuracy to the performance of minority classes, each only summarizes classifier performance at a single decision threshold: The predicted probability of an image belonging to a class is converted to a label only when it surpasses a fixed, and often arbitrarily defined threshold [66]. To overcome this, we generated precision-recall curves at every decision threshold to visualize their trade-off—in other words, the relationship between the fraction of correctly predicted true positives (predictive positive value) and the true positive rate [67]. Precision-recall curves are robust for imbalanced classification because they are unaffected by the increasing true negatives after labels are binarized [68]. To summarize classifier performance for each class across every decision threshold, we computed the average precision of each class (4), where ${R}_{n}$ and ${P}_{n}$ are recall and precision at the nth threshold, respectively [48].

4 $$Average\, Precision= \sum_{n}{(R}_{n}- {R}_{n-1}) {P}_{n}$$

Average precision is analogous to a non-linear interpolation of the area under each precision-recall curve (AUC-PR) [69]—as a rank measure, the AUC is closely related to statistical separability between classes [64, 67]. For a specific class, the performance baseline when evaluating AUC-PR is defined by the ratio of positives $\left(P\right)$ to negatives $(N)$ in the test set $y= \frac{P}{P+N}$, and is equal to the probability of a positive example being correctly classified over a negative example [68]. The baseline is therefore different for each class.

## Results

### Holographic data

**Fig. 2:**
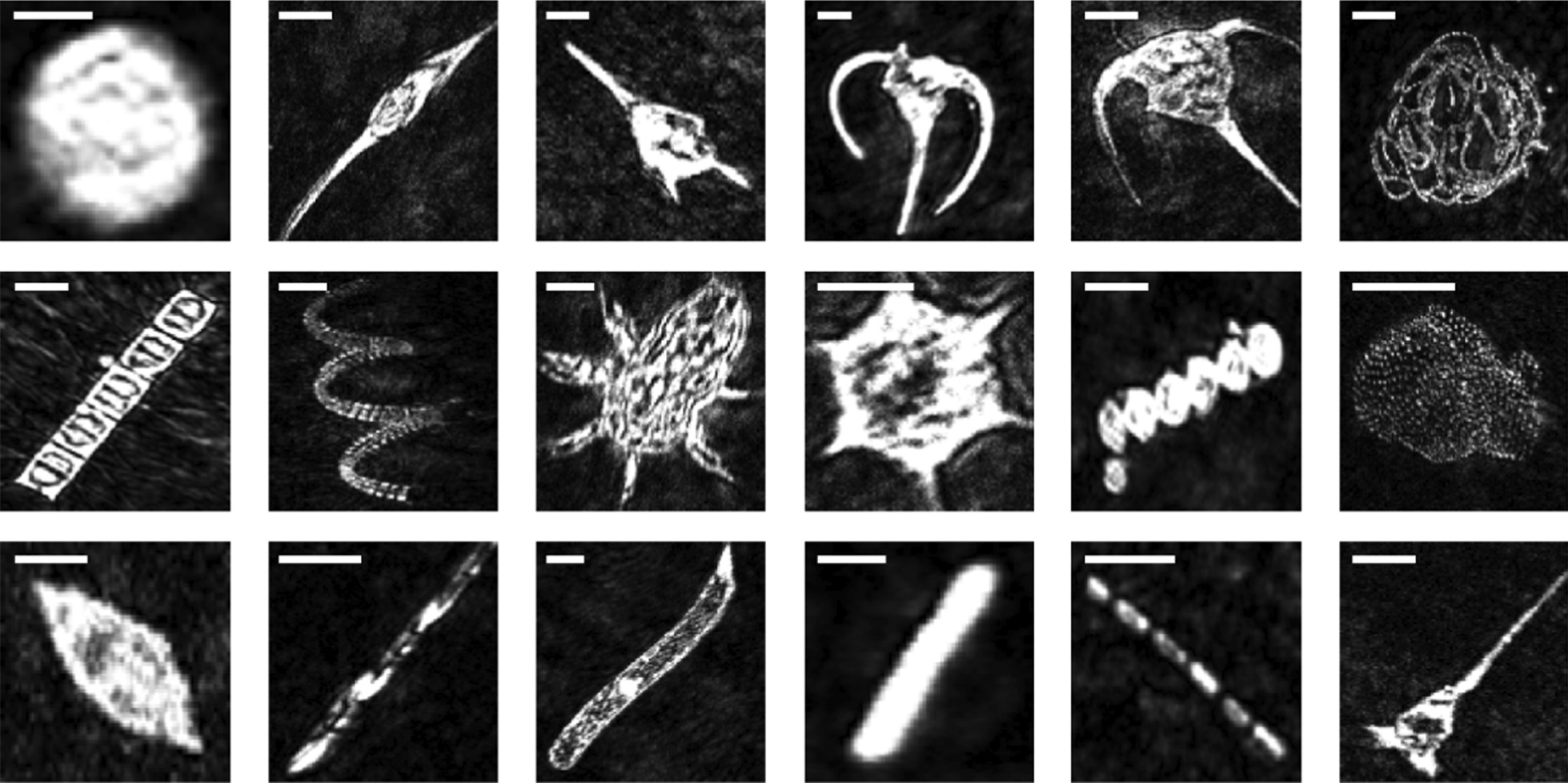
Amplitude images reconstructed and detected from specific focal planes for each plankton class. From top left to lower right: *Alexandrium tamarense, Ceratium fusus*, *Ceratium lineatum*, *Ceratium longpipes*, *Ceratium* sp., *Chaetoceros socialis, Chaetoceros* straight, *Chaetoceros* sp., Crustacean, *Dictyocha speculum*, *Melosira octagona, Parvicorbicula socialis*, *Prorocentrum micans*, *Pseudo-nitchzia arctica, Rhizosolenia setigera,* Rods, *Skeletonema costatum*, Tintinnid*.* All images are segmented to 128 × 128 pixels and scale bars represent 50 µm

In total, > 17,000 holograms comprising > 70 GB of data were produced from our samples. Reconstructed by Octopus software, holograms had the highest intensity in the central axis which attenuated at the hologram edges (Fig. 1). Hologram intensity was reconstructed in the order of eight milliseconds for a 2048 × 2048 hologram. The numerical holograms reconstruction, ROI clustering, and autofocusing that compose our multi-stage detection steps generated 3826 in-focus plankton objects from 19 classes (Fig. 2). In total, the full workflow amounted to approximately 44 h of computational time dominated by in-focus detection (> 95%), and the remainder by classification. Six classes were generated from the environmental samples including *C. fusus*, *C. lineatum*, *Ceratium* sp., Crustaceans, *P. socilais*, and Tintinnids. The remaining classes derived from monoculture and represented individual plankton species. In total, the environmental classes were less abundant than classes derived from pure cultures. The size of plankton objects ranged from 20 to 640 μm, with the majority smaller than 200 μm and belonging to microplankton (Table 1). The classes proved highly imbalanced with the greatest difference between mesoplankton Crustaceans containing 13 images, and the microplankton dinoflagellate *P. micans* containing 1074 images (Table 1). After augmentation, the CNN training data contained 7215 samples which when subdivided into stratified folds contained 5772 images for training and 1443 images for validation.

### Overall classification

The classification source code is publicly available on Github [70]. The feature extraction and retraining methods produced indistinguishable performance results across classification metrics, so we will consider only the feature extraction results here. For feature extraction, the overall classification performance based on the accuracy, precision, recall, and F1-score are reported in Table 2. The InceptionV3 model achieved the lowest precision values at 83% and F1-score of 81%. All the remaining three models performed comparably reaching precision scores > 88%, and F1-scores > 87%. The Xception model consistently outperformed every other to achieve precision and F1-scores of 89%. The underlying classification performance for each taxon is described below by their AUC-PR. Each model clearly achieved maximum precision, recall, and F1-scores quickly—in five or fewer epochs—while the mean and standard deviation for predictions across epochs was generally low (< 2.5%). The log loss error showed similar model behaviour overall, with error minima in fewer than five epochs and Xception obtaining the lowest error.

**Table 2:** Average performance of each model across folds for each threshold metric on the test set

		Threshold metrics (%)			
	Model	Accuracy	Precision	Recall	F1-Score
Feature extraction	VGG16	88.2 ± 1.2	88.4 ± 1.5	88.1 ± 0.9	87.8 ± 1.0
	InceptionV3	82.2 ± 1.8	83.7 ± 2.4	81.1 ± 2.2	81.7 ± 1.4
	ResNet50V2	88.2 ± 1.1	88.6 ± 1.3	88.1 ± 07	87.9 ± 0.9
	Xception	90.1 ± 1.6	89.8 ± 0.9	90.7 ± 0.4	89.8 ± 0.7

### Taxa-level classification

The AUC-PR values for each class are reported in Table 3. The precision-recall curves for each model (See Additional file 1: Figures S4–7) broadly showed that the highest AUC-PR values and therefore the 11 highest ranked classes included the dinoflagellates *A. tamaranse* and all four *Ceratium* taxa, along with diatoms for all three *Chaetoceros* taxa and *M. octagona,* the silicoflagelle *D. speculum*., and our noise class. Both Xception and ResNet50 ranked the rarest class of Crustaceans highly. As the best classifier, Xception even ranked rare taxa *C. lineatum* (0.91) and Crustacean (0.86) higher than the *Chaetoceros* straight morphotype (Fig. 2), despite containing less than a seventh of examples. Classification performance deteriorated for the remaining seven taxa to ranking only marginally better than random for the choanoflagellate *P. socialis*, and the diatoms including *P. arctica*. and *S. costatum*, as well as the Rods morphotype and the ciliate Tintinnids. The dinoflagellate *P. micans* was the only taxa that was unanimously ranked worse than random in each model—that is, AUC-PR values below their class baseline—despite it containing nearly three times as many examples as the next most abundant class. No clear difference in classification performance occurred between size classes.

**Table 3:** Area under the precision-recall curves calculated using average precision for each class

Class	AUC-PR			
	VGG16	InceptionV3	ResNet50V2	Xception
*Alexandrium tamarense*	0.97	0.85	0.96	0.98
*Ceratium fusus*	0.88	0.55	0.78	0.89
*Ceratium lineatum*^*a*^	0.76	0.60	0.76	0.91
*Ceratium longpipes*	0.97	0.93	0.98	0.99
*Ceratium* sp.	0.79	0.59	0.85	0.92
*Chaetoceros socialis*	0.98	0.96	0.99	0.99
*Chaetoceros* straight	0.80	0.61	0.77	0.84
*Chaetoceros* sp.	0.93	0.83	0.96	0.98
Crustacean^a^	0.56	0.30	0.84	0.86
*Dictyocha speculum*	0.98	0.88	0.97	0.99
*Melosira octagona*	0.98	0.92	0.97	0.98
Noise	0.96	0.87	0.94	0.98
*Parvicorbicula socialis*^*a*^	0.01	0.01	0.01	0.01
*Prorocentrum micans*^*a*^	0.19	0.21	0.20	0.16
*Pseudo-nitchzia arctica*^*a*^	0.02	0.01	0.03	0.03
*Rhizosoenia setigera*	0.05	0.04	0.04	0.04
Rods	0.19	0.13	0.14	0.14
*Skeletonema costatum*	0.06	0.07	0.05	0.06
Tintinnid^a^	0.01	0.01	0.01	0.02

^a^Indicate rare classes with < 25 examples in the training set

## Discussion

This work demonstrates the usefulness of DIHM equipped with a workflow for volumetric hologram reconstruction, objection detection and autofocusing to classify plankton images using off-the-shelf CNNs. In general, plankton size did not obviously affect classification, but the sharpest images and most resolvable features were ranked higher, except for the dinoflagellate *A. tamarense*, which was likely well recognized as the only visually circular species in the dataset. In the highly ranked dinoflagellates, apical and antapical horns in *C. fusus* and *C. lineatum* and the spines in *C. longpipes* and *Ceratium sp.* resolved clearly and were conspicuous features. The dinoflagellate *P. micans* was poorly resolved and classified, it is possible that the small cell size (< 100 μm) limited any detection of its thecal plates or small (< 10 μm) apical spine [71]. For the diatoms, the chained *C. socialis*, *Chaetoceros* sp., and *M. octagona* were all distinct from each other with colonies, spirals, and straight chains that likely contributed to their reliable classification. More broadly, many chained objects showed a discernible interstitial space between cells which was especially distinct in *Chaetoceros* and *S. costatum* (Fig. 2), although the setae of the *Chaetoceros* classes was rarely visible. Comparatively, the poorly ranked diatoms *S. costatum, R. setigera*, and *P. arctica* all lacked morphological definition. Similar to the WHOI plankton dataset [40], the small sized choanoflagellate *P. socialis* only displayed colonies of flame bulbs and the silica loricae and flagellum cannot be seen—likely explaining its unanimously poor ranking by each CNN.

The complex morphology of plankton also presents a problem of image scale: The features available for detection in this study were limited to those that remained after objects were segmented to 128 × 128 scale. These image sizes are different from the ImageNet images used to train each CNN—VGG16 and ResNet50 were trained on 224 × 224 images and InceptionV3 and Xception were trained on 229 × 229 images. This suggests encouraging transferability to our holographic plankton images. Although scaling effectively normalizes the wide variety of features and explicitly retains scale invariant features, imaged plankton features can obviously vary with size, and therefore scale invariant features only partially describe the spatial composition of any object [72]. Segmenting objects at multiple scales could capture scale-variant features, but examples of scale-variant detection are less common. Artist attribution is an example of a complex classification task where multi-scale images (256, 512, 1024, 2048 pixels) systematically improved CNN predictions using both coarse and fine grain features of digitized artworks belonging to the Rijksmuseum, at the Netherlands State Museum [73]. But currently, multi-scale CNNs lose scale invariant features that otherwise emerge during scaling and augmentation, and these features are not guaranteed to emerge during convolutional feature extraction. Further research on scale-variant feature detection could overcome this limitation and help identify the diversity of plankton features that are more or less resolvable at different scales.

Holography has certain technical challenges for capturing high-quality plankton features, owing first to the need for numerical reconstruction of a sample volume, followed by object detection and autofocusing. In assessing the HoloSea, Walcutt et al. [41] observed two notable biases underlying particle size and density estimates, including the attenuated light intensity from the point source, both radially and axially across the sample volume and secondly, that foreground objects inevitably shade the volume background. Although this study is concerned with classification, both biases are present in this study. Several modifications offered by Walcutt et al. [41] apply here: Adjusting the point source-to-camera distance to expand sample space illumination and create a more uniform light intensity, scaling object detection probability based on pixel intensity, and local adaptive thresholding to improve ROIs detection consistency at the dimmed hologram edges—as opposed to the fixed, global thresholding algorithm used here. Because objects are less likely to be detected at the hologram edges, only a fraction of the particle field is consistently imaged. The total volume imaged, calculated as the product of the number of holograms and the volume of each hologram (maximally 0.1 mL), should be corrected by the actual illuminated proportion of the sample volume: For the HoloSea, Walcutt et al. [41] empirically derived the working image volume at 0.063 mL per hologram. The digital corrections are likely simpler and should be implemented in future quantitative assessments, unless the increasing ability of deep learning algorithms in holographic reconstruction, enhancing depth-of-field and autofocusing can outperform instrument-specific corrections [17]. Nonetheless, holography opens new opportunities for high-throughput volumetric image analysis and the robust modular casings of DIHM—which operate in the abyssopelagic zone (~ 6000 m) [74] and High Arctic springs [75]—make for versatile instruments to deploy in oceanic environments.

Classification tasks for almost every image domain have greatly improved with transfer learning [76], including for plankton [77]. With a transfer learning approach, our results show good classification performance for multiple groups of abundant micro and mesoplankton—encompassing the size spectra (5–50 µm) that microbial eukaryotic diversity peaks [78]. Classification performance was also high for several rare taxa including Crustaceans, *C. fusus*, *C. lineatum*, and *Ceratium* sp., all of which contained fewer than 50 training examples. Publicly shared datasets like ImageNet have been central for classification benchmarks, increasing training examples for a wider recognition of features within and across imaging modes and minimizing the imbalance of class distributions in small and large datasets [79]. For plankton, open access datasets such as the In Situ Ichthyoplankton Imaging System [5] dataset shared through the Kaggle's National Data Science Bowl competition, and the WHOI dataset captured by the Imaging Flow Cytobot [4] are important starting points. But both image modes are quite different from holographic images: To improve transferability of feature recognition, an open database specific to the holographic domain could promote wider use and shrink the gap between its high-throughput image production and analyses. To that end, the holographic plankton images used here will be publicly available in the Cell Image library (See ‘Availability of data and materials’).

Although the primary concern of this work is detection and classification from holographic images, generalizing classifiers to unseen plankton populations remains challenging [35]. Plankton vary widely and are invariably observed unevenly. However rare plankton classes can be important and removing them from datasets (e.g., [34, 65]) is not desirable if imaging instruments are to be maximally effective in sampling the plankton community. Ballast water quality testing, for example, relies on presence-absence of rare, invasive taxa [80]. The proper classifier evaluation is in performance on the original imbalanced datasets, not how certain performance measures can be tuned by synthetically manipulating class balances [81]. As an alternative, optimizing decision thresholds in precision-recall curves for each class has seen revived interest, and benefits from bypassing the generated biases in common oversampling methods [82]. For evaluating classifiers of imbalanced plankton datasets, we encourage wider use of ranking metrics like AUC-PR, which summarize the trade-offs of any particular metric at every decision threshold and appear rarely used in plankton classification tasks (e.g., [83, 84]).

In machine learning, quantification is increasingly separated from classification as a different, and altogether more challenging learning task; several quantification approaches are reviewed in González et al. [85]. For in-situ plankton imaging systems, classification algorithms do not account for shifting class distributions across samples, false positive rates acquired during model training, and because most plankton studies aim to estimate total group abundance across observations in space, or through time, the learning problem then becomes at the level of the sample, not the individual image [35]. Although any classifiers false positives can be corrected for (e.g., [86]), a generalizable classifier would contain robust sample-level error, not at the taxon level [35]. The features learned by CNNs for classification, similar to those described here, can be used for plankton quantification. González et al. [87] input high-level features from pre-trained CNNs into quantification algorithms to estimate plankton prevalence throughout more than six years of the Martha’s Vineyard time series collected by the Imaging Flow Cytobot and showed high correspondence—even approaching perfect—between probabilistic quantifiers and ground-truth estimates even in rare taxa (< 1 mL^−1^). These results are encouraging that even imperfect quantifiers can deliver biologically meaningful estimates of plankton.

## Conclusion

This work integrates a simple and deployable high-throughput holographic microscope with autofocused object detection and state-of-the-art deep learning classifiers. The combined high-throughput sampling and digital image processing of the HoloSea shows its ability to produce and reconstruct sharp images of important plankton groups from both culture and environmental samples, although some further optical corrections are desirable. Classifying a wide-ranging plankton classes, both rare and abundant, the pre-trained CNNs showed compelling baselines through rapid learning and complex feature recognition despite the starkly different holographic image domain. Overall, this ensemble of tools for holographic plankton images can confidently separate and classify the majority of our micro-mesoplankton classes. With the exception of a small dinoflagellate and choanoflagellate with poorly resolved features, classification performance was unaffected by plankton size.

Holographic microscopes are well suited for volumetric sampling in aquatic ecosystems and the relatively simple in-line microscope configurations, comparable to the model used here, can be modified for robust designs to deploy in harsh environments. These advantages allow in-line holographic microscopes to be towed, attached to conventional CTD rosettes, or stationed in situ for continuous monitoring. Moreover, the recent achievements in holographic reconstruction and image processing allow micrometer resolution from high-throughput instruments. Achieving real-time data interpretation remains unfeasible, but the rapid sampling capacity of holography leaves automatic classification, although improved, an outstanding challenge.

We contribute a publicly available dataset to improve CNN transferability and enhance benchmarks for plankton classification. The improvements in holographic hardware and digital capacity argues for wider use in aquatic microbial ecology and more broadly, its high-throughput potential and data-rich images warrants wider adoption in cell imaging tasks.

## Supplementary Information

**Additional file 1: Figure S1.** Left to right, distribution of taxa abundance for training set—where the distribution ratios are maintained during stratified cross validation—and the test set. **Figure S2.** Four classified noise objects with no resolvable features. **Figure S3.** Network architecture for basic CNN. **Figure S4.** Precision-recall curves of the InceptionV3, with iso-curves for their harmonic mean F1-score, and the area under the curve (AUC-PR). **Figure S5.** Precision-recall curves of the InceptionV3, with iso-curves for their harmonic mean F1-score, and the area under the curve (AUC-PR). **Figure S6.** Precision-recall curves of the InceptionV3, with iso-curves for their harmonic mean F1-score, and the area under the curve (AUC-PR). **Figure S7.** Precision-recall curves of the Xception model for each class, with iso-curves for their harmonic mean F1-score, and the area under the curve (AUC-PR). **Table S1.** The reference paper of four CNNs, their convolutional layers, the weighted layers that are changed during backpropagation, and broad overview of their key features. **Table S2.** Total time and memory expended for training and evaluating each model averaged for feature extraction and fine tuning. **Table S3.** Average performance of each model for each threshold metric on the test set for each fold.

## Data Availability

The dataset supporting the conclusions of this article is available in the Cell Image library repository [Training data: http://cellimagelibrary.org/groups/53406; Test data: http://cellimagelibrary.org/groups/53362].

## Abbreviations

AUC: Area under the curve
AUC-PR: Area under precision-recall curve
CMOS: Complementary metal oxide semiconductor
CNN: Convolutional Neural Network
DBSCAN: Density Based Spatial Clustering with Applications of Noise
DIHM: Digital in-line holographic microscope

## Footnotes

1 http://4-deep.com/

2 http://4-deep.com/products/octopus-software/

3 http://4-deep.com/products/stingray-software/
