# Supplementary material for: Plankton classification with high-throughput submersible holographic microscopy and transfer learning
Source: BMC Ecol Evol. 2021 Jun 16;21:123. doi: 10.1186/s12862-021-01839-0 (PMC8207568; doi:10.1186/s12862-021-01839-0)
Supplement: Supplementary file 1 — Additional file 1: Figure S1. Left to right, distribution of taxa abundance for training set—where the distribution ratios are maintained during stratified cross validation—and the test set. Figure S2. Four classified noise objects with no resolvable features. Figure S3. Network architecture for basic CNN. Figure S4. Precision-recall curves of the InceptionV3, with iso-curves for their harmonic mean F1-score, and the area under the curve (AUC-PR). Figure S5. Precision-recall curves of the InceptionV3, with iso-curves for their harmonic mean F1-score, and the area under the curve (AUC-PR). Figure S6. Precision-recall curves of the InceptionV3, with iso-curves for their harmonic mean F1-score, and the area under the curve (AUC-PR). Figure S7. Precision-recall curves of the Xception model for each class, with iso-curves for their harmonic mean F1-score, and the area under the curve (AUC-PR). Table S1. The reference paper of four CNNs, their convolutional layers, the weighted layers that are changed during backpropagation, and broad overview of their key features. Table S2. Total time and memory expended for training and evaluating each model averaged for feature extraction and fine tuning. Table S3. Average performance of each model for each threshold metric on the test set for each fold. [file 12862_2021_1839_MOESM1_ESM.docx]

**Additional Material: Plankton classification with high-throughput submersible holographic microscopy and transfer learning**

Liam MacNeil^1^, Sergey Missan^2^, Junliang Luo^3^, Thomas Trappenberg^3^, Julie LaRoche^1^

^1^Biology Department, Dalhousie University, 1355 Oxford Street, Halifax, Nova Scotia, Canada, B3H 4J1

^2^4Deep inwater imaging, 71 Appaloosa Run, Hammonds Plains, Nova Scotia, Canada, B4B 0G2

^3^Department of Computer Science, Dalhousie University, 6050 University Avenue, Halifax, Nova Scotia, B3H 4R2

Corresponding Author: Liam MacNeil; [L.macneil@dal.ca](mailto:L.macneil@dal.ca)

***Methods***

**Holographic Image Dataset**


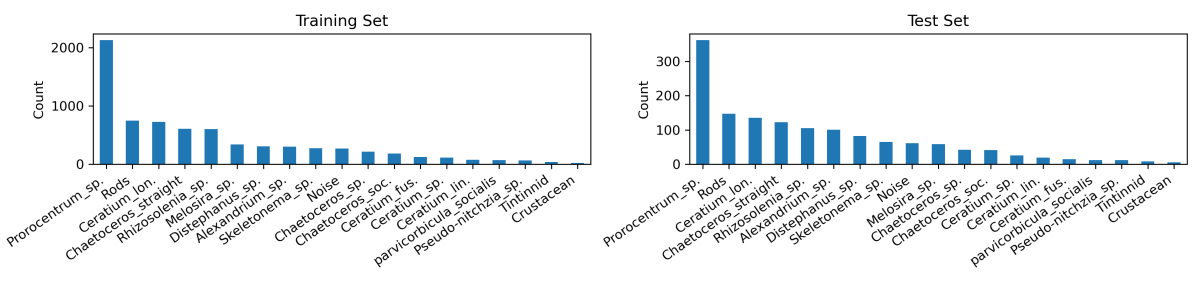


Fig. S1 Left to right, distribution of taxa abundance for training set— where the distribution ratios are maintained during stratified cross validation— and the test set.


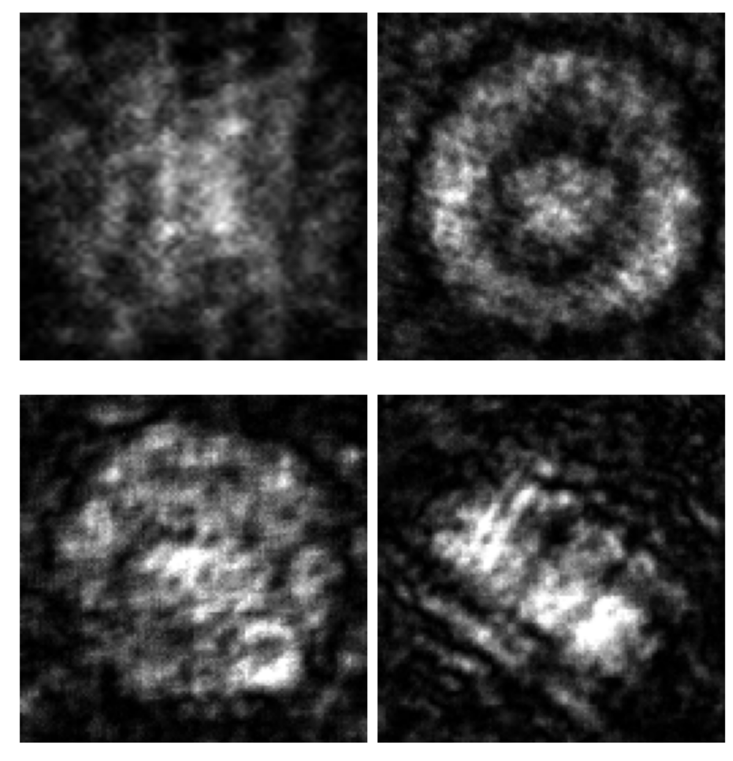


Fig. S2 Four classified noise objects with no resolvable features. Image artefacts are a challenge for any imaging system operating in real time, where both the imaging mode and environment can create non-biological objects.

**Convolutional Neural Networks**

Table S1. The reference paper of Four CNNs, their convolutional layers, the weighted layers that are changed during backpropagation, and broad overview of their key features.

| Model | Reference | Convolutions | Trainable Parameters | Description |
| --- | --- | --- | --- | --- |
| VGG16 | Simonyan and Zisserman (2014) | 13 | 138,357,544 | Stacked sequence of 3×3 convolution filters and max pooling layers. Three fully connected layers compile features. Runner up in ILSVRC 2014. |
| InceptionV3 | Szegedy *et al.* (2016) | 48 | 23,851,784 | Parallel convolution filters and pooling layers to extract features with different filter sizes (multiple sized receptive fields) known as depth wise separable convolutions. Parallel filters recognize features of multiple sizes within a layer. The Inception module (GoogLeNet) winner in ILSVRC 2014. |
| ResNet50V2 | He *et al.* (2016) | 50 | 25,613,800 | ResNets composed of sequential layers in blocks, with identity connections between blocks that sum the output of each block with the previous block. These shortcut connections are most important during learning⎯ the backpropagation algorithm⎯ where error gradients can be conveyed to the earliest layers. ResNets overcame vanishing gradients in learning deeper, more complex models, winning ILSVRC 2015. ResNetV2 only applies non-linearity (ReLu) before convolutions within a block. |
| Xception | Chollet (2017) | 71 | 22,910,480 | Extreme Inception modules perform 1×1 convolution before depthwise separable convolutions with shortcut connections between convolution blocks analogous to ResNets. The top accuracy from the ILSVRC outperforms VGG16, InceptionV3, and ResNet152 |

Fig. S3 Network architecture for basic CNN. The inputs are the resized (128×128) images containing in-focus objects. CNN schematic created in <http://alexlenail.me/NN-SVG/LeNet.html>

**Validation Measures**

Data skew and asymmetric learning has proven imbalanced classification as an especially challenging problem within deep learning (Johnson and Khoshgoftaar, 2019). Evaluating imbalanced problems by illustrating performance metrics at every decision threshold parses the individual trade-off between true and false positives and is a more robust evaluation for classifiers. In particular, PR curves are sensitive to large changes in false positive rates due to the direct comparison of true and false positives that compose precision (Jeni *et al.* 2013). This is an advantage over true and false positive rates across every decision threshold involved in a Receiving Operating Characteristic (ROC) (Fawcett, 2006): the large amount of true negatives created by binarizing classes in one vs all in imbalanced problems can overcome model sensitivity to false positives and produce over optimistic ROC plots and the area under each class curve (Jeni *et al.* 2013). In plankton classification, false positives are important where presence-absence data are reported, or when interpreting datasets across time or space with high fidelity. As alternative model summary statistic, we also calculated the weighted mean of precision scores at each decision threshold (Boyd *et al.* 2013). Lastly, we recorded the probabilistic model error defined by a log loss (cross entropy) function, measuring the divergence of predicted class probabilities from their true label (Janocha and Czarnecki, 2017).

***Results***

Table S2. Total time and memory expended for training and evaluating each model averaged for feature extraction and fine tuning.

| Model | Time (minutes) | Memory Consumption (GB) |
| --- | --- | --- |
| VGG16 | 83 | 3.61 |
| InceptionV3 | 79 | 0.83 |
| ResNetV2 | 74 | 1.28 |
| Xception | 71 | 1.24 |

Table S3: Average performance of each model for each threshold metric on the test set for each fold. See supplementary for the mean and standard deviation on training and validation sets for each stratified fold.

|  |  | Threshold Metrics (%) | | | |
| --- | --- | --- | --- | --- | --- |
|  | Model | Accuracy | Precision | Recall | F1-Score |
|  | VGG16 | 88.2 ± 0.6 | 88.4 ± 0.5 | 88.1 ± 0.6 | 87.8 ± 0.9 |
| Feature Extraction | InceptionV3 | 79.8 ± 1.2 | 77.2 ± 2.0 | 82.6 ± 0.7 | 78.9 ± 1.4 |
|  | ResNetV2 | 88.2 ± 0.8 | 88.6 ± 0.7 | 88.1 ± 07 | 87.9 ± 0.9 |
|  | Xception | 90.1 ± 0.6 | 89.8 ± 0.9 | 90.7 ± 0.4 | 89.8 ± 0.7 |
| Retraining Deeper Layers | VGG16 | 88.7 ± 0.3 | 88.5 ± 0.3 | 88.8 ± 0.6 | 88.3 ± 0.4 |
|  | InceptionV3 | 79.7 ± 0.09 | 77.8 ± 1.9 | 81.9 ± 0.03 | 79.0 ± 1.2 |
|  | ResNetV2 | 87.9 ± 1.0 | 87.6 ± 1.3 | 88.6 ± 0.7 | 87.5 ± 1.1 |
|  | Xception | 90.2 ± 1.0 | 89.9 ± 2.0 | 90.8 ± 1.0 | 90.0 ± 1.8 |


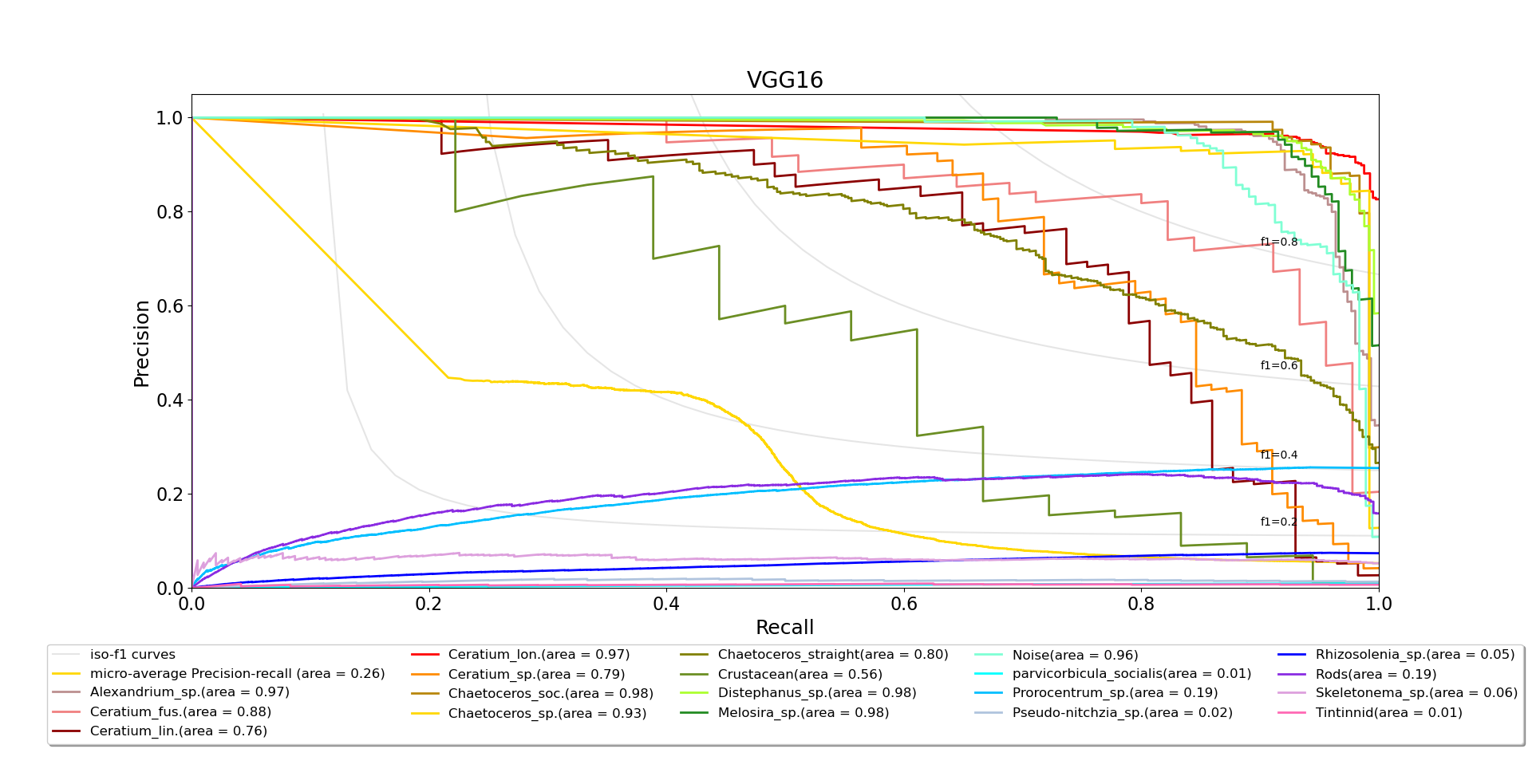


Fig. S4 Precision-recall curves of the InceptionV3, with iso-curves for their harmonic mean F1-score, and the area under the curve (AUC-PR).


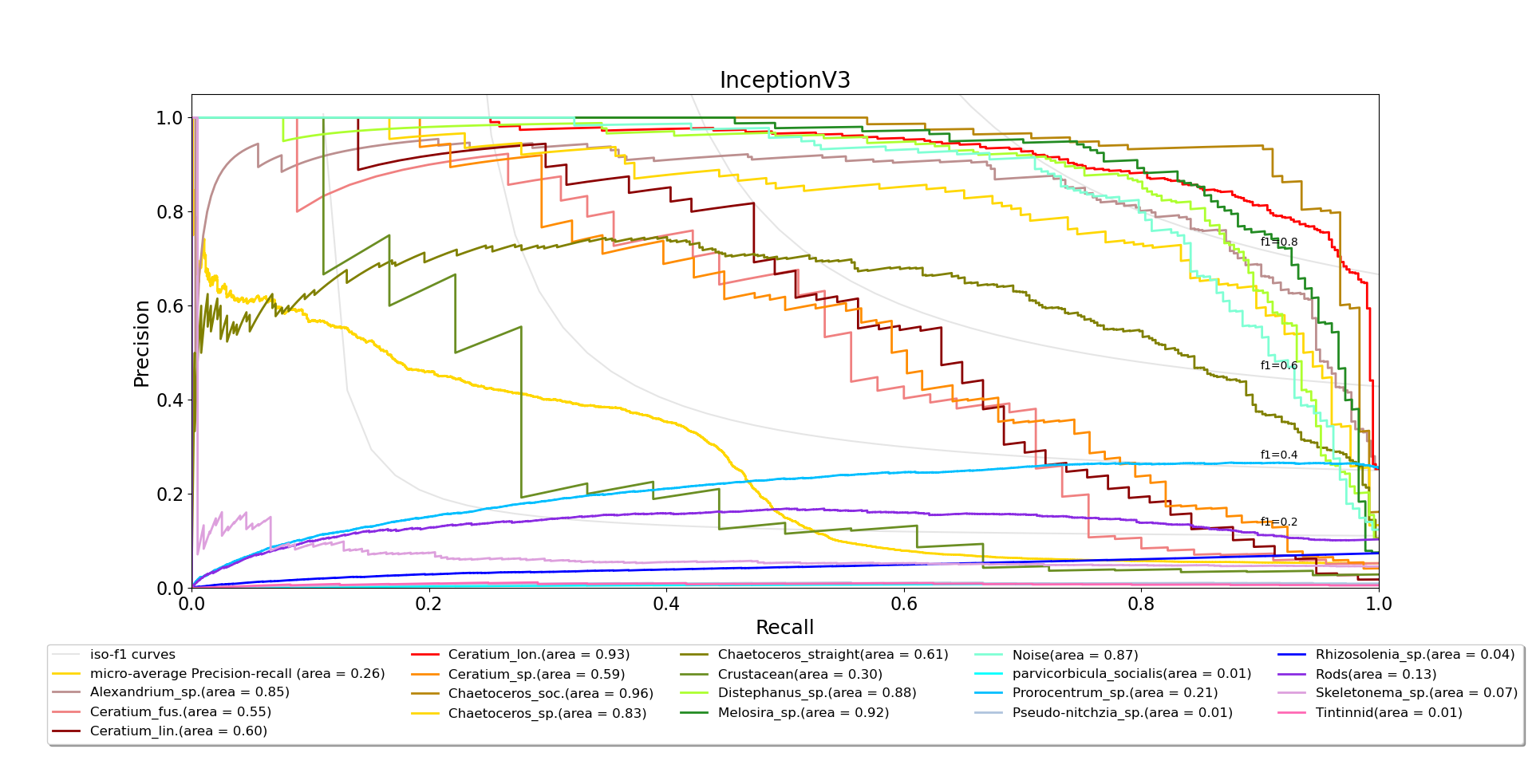


Fig. S6 Precision-recall curves of the InceptionV3, with iso-curves for their harmonic mean F1-score, and the area under the curve (AUC-PR).


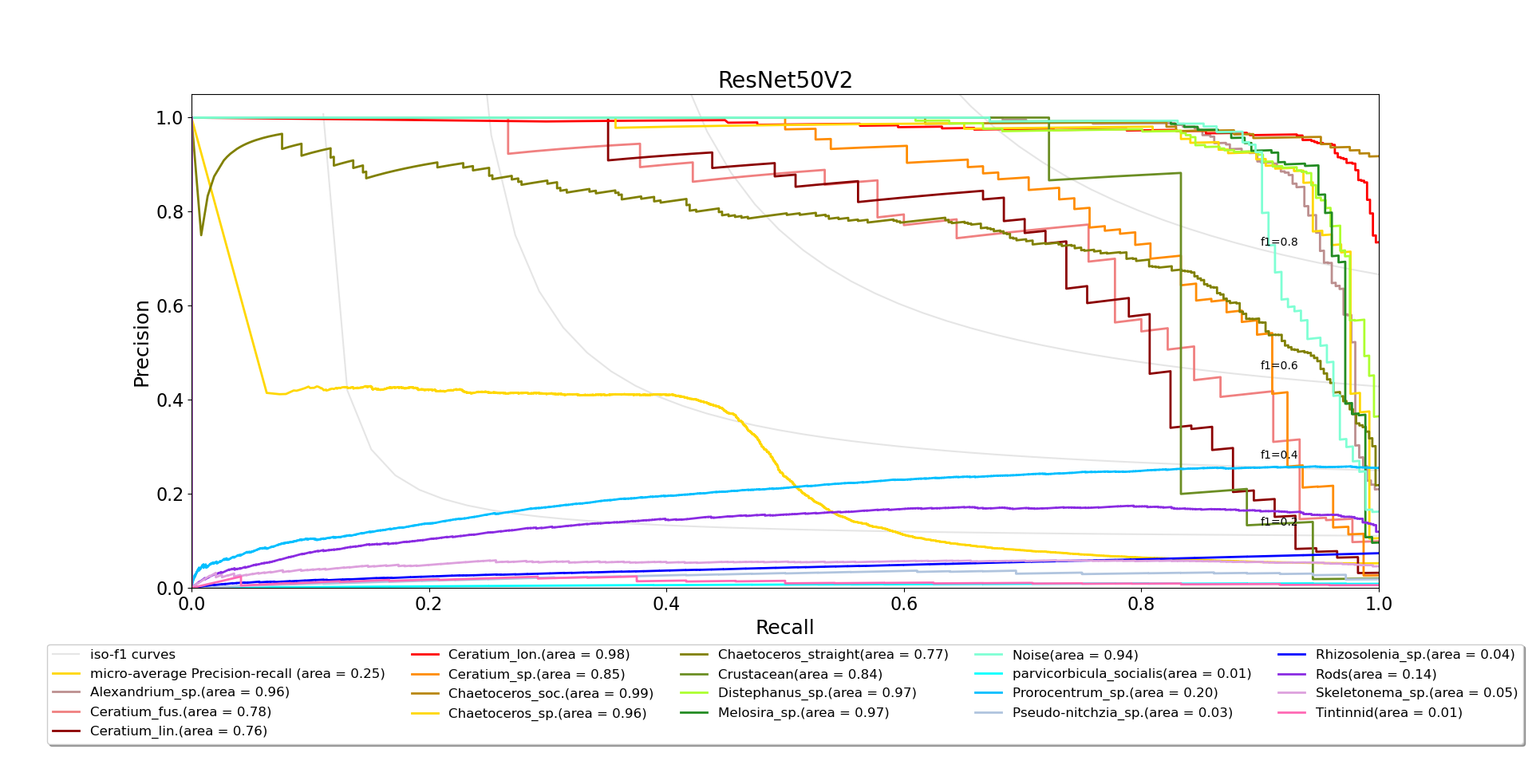


Fig. S5 Precision-recall curves of the InceptionV3, with iso-curves for their harmonic mean F1-score, and the area under the curve (AUC-PR).


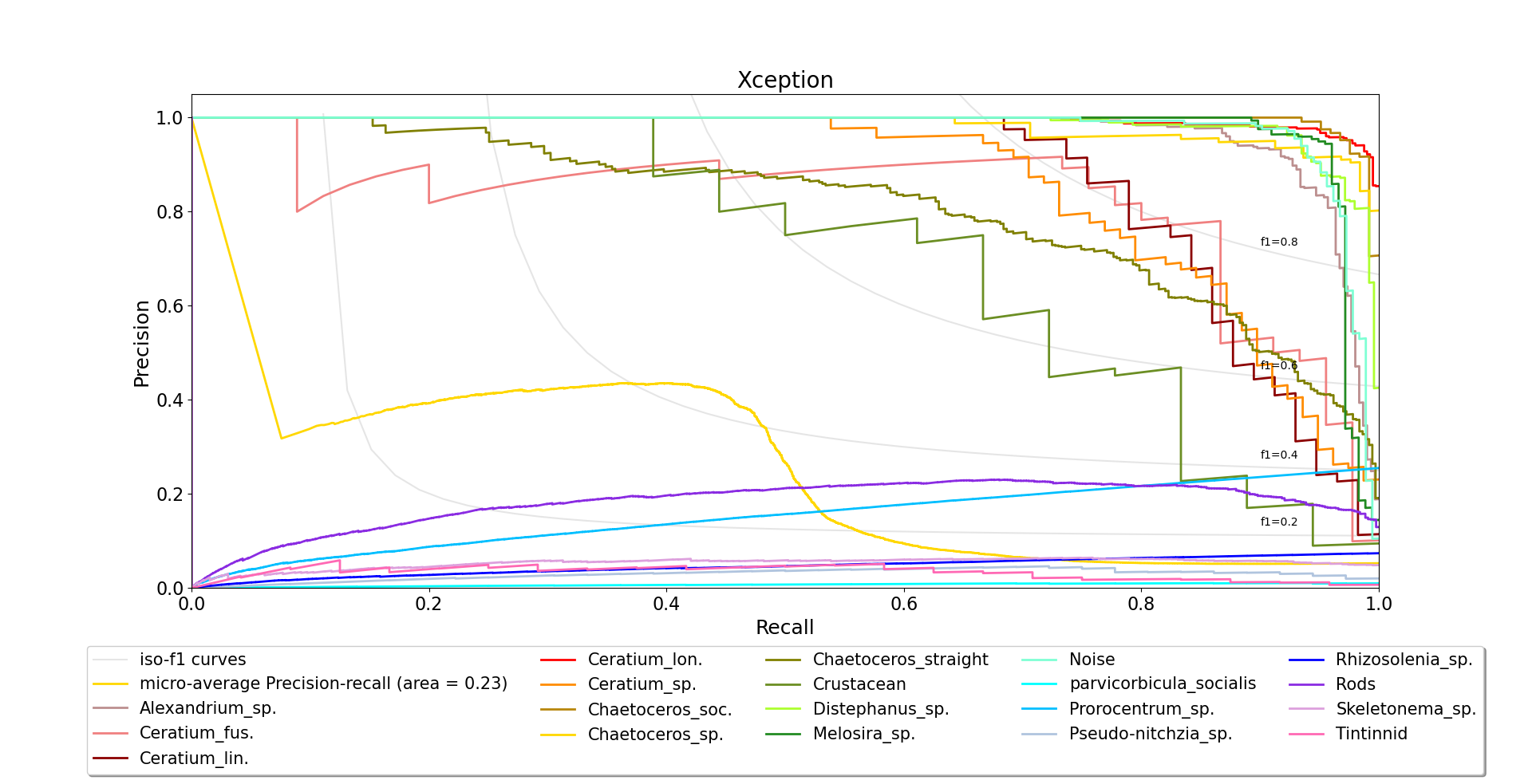
Fig. S7 Precision-recall curves of the Xception model for each class, with iso-curves for their harmonic mean F1-score, and the area under the curve (AUC-PR).

***References***

Fawcett, T. An introduction to ROC analysis. Pattern Recognit Lett. 2006; 27(8): 861–874. <https://doi.org/10.1016/j.patrec.2005.10.010>

Janocha K, Czarnecki WM. On Loss Functions for Deep Neural Networks in Classification. arXiv preprint. 2017; doi: [arXiv:1702.05659v1](https://arxiv.org/abs/1702.05659v1).

Jeni LA, Cohn JF, De La Torre F. Facing Imbalanced Data—Recommendations for the Use of Performance Metrics. 2013 Humaine Association Conference on Affective Computing and Intelligent Interaction. 2013; 245–251. <https://doi.org/10.1109/ACII.2013.47>

Johnson JM, Khoshgoftaar TM. Survey on deep learning with class imbalance. J Big Data. 2019; 6(1): 27. <https://doi.org/10.1186/s40537-019-0192-5>.
